# Supplementary material for: Effect of kinematics on ground reaction force during single-leg jump landing in children: a causal decomposition approach in jumpers and non-jumpers
Source: PeerJ. 2024 Nov 25;12:e18502. doi: 10.7717/peerj.18502 (PMC11604040; doi:10.7717/peerj.18502)
Supplement: Supplemental Information 2 — Strength of causality between non-jumpers vs jumpers and between joints. [file peerj-12-18502-s002.docx]

| **Joint** | **Mean Difference**  **of RCS** | **95% Confidence Interval for Difference** | **P-value** | ***d*** |
| --- | --- | --- | --- | --- |
| Hip | 0.016 | -0.020 to 0.053 | 0.359 | 0.38 |
| Knee | 0.006 | -0.030 to 0.042 | 0.739 | 0.14 |
| Ankle | **-0,043** | **-0.077 to -0.008** | ***0.017** | **1.03** |

**Supplementary table 1.** Strength of causality between non-jumpers vs jumpers.

Relative causal strength (RCS). *p < 0.05. Statistical significance is marked in bold. Effect sizes are indicated by Cohen's d (*d*).

**Supplementary Table 2.** Strength of causality between joints in non-jumpers and jumpers.

|  | **Joint comparisons** | | **Mean Difference of RCS** | **95% Confidence Interval for Difference** | **P-Value** | ***d*** |
| --- | --- | --- | --- | --- | --- | --- |
| No-jumpers | Hip | Knee | 0.041 | -0.007 to 0.88 | 0.111 | 0.69 |
|  |  | Ankle | **0.067** | **0.020 to 0.113** | ***0.004** | **0.91** |
|  | Knee | Ankle | 0.026 | -0.016 to 0.68 | 0.383 | 0.57 |
| Jumpers | Hip | Knee | 0.030 | -0.012 to 0.72 | 0.228 | 0.50 |
|  |  | Ankle | 0.007 | -0.034 to 0.49 | 1.000 | 0.15 |
|  | Knee | Ankle | -0.023 | -0.060 to 0.14 | 0.379 | 0.38 |

Relative causal strength (RCS). *p < 0.05. Statistical significance is marked in bold. Effect sizes are indicated by Cohen's d (*d*).
