# Supplementary material for: Effect of kinematics on ground reaction force during single-leg jump landing in children: a causal decomposition approach in jumpers and non-jumpers
Source: PeerJ. 2024 Nov 25;12:e18502. doi: 10.7717/peerj.18502 (PMC11604040; doi:10.7717/peerj.18502)
Supplement: Supplemental Information 3 [file peerj-12-18502-s003.docx]

**Single-leg landing in children**

PROCEDURE

*Participants*

Three different groups (typically jumping sports (a) gymnastics, (b) voleyball, & (c) control), each of 10 children, are involved in the study (n = 30). Sociodemographic and anthropometric data should are similar (e.g., nationality, PE hours/week, age and BMI). Participants’ age is from 8 to 12 (both included) and sport participants should have at least one year of experience in the practice of the sport.

Common jumping sports refer to those disciplines in which the jumping action, and consequently landing, is presented frequently. Taking into account that the characteristics of every discipline can make that the type and nature of jumping is different, two different type of sports are recognized in the organized sport groups:

- Gymnastic (n = 10) (wherein the jump every time finish with the two legs and during landing the aim is to maintain as quite as possible).
- Volley ball (n = 10) (where the jump finish with either one or two legs but during athlete can move).

*Data acquisition*

A system composed by 8 motion capture cameras and one force platform, both system synchronized, were used for data collection. Every child performed a total of 30 drops with single-leg landing (SLL) using the preferred leg, that is, 10 trials from 25 cm with preferred lower limb. After each trial children must maintain balance for 10 seconds for. A target located at the child’s chin 2m opposite of the force platform was provided.

Every trial started from a specific height with the child in a stance position and the hands located in the hip under the superior iliac crest. The structure for different height was located 10 cm behind the force platform. From a self-decision the child made a step with the preferred leg landing inside the force platform. All children were encouraged to land with one leg maintaining the stability for 10 seconds with the knee of the free leg in approximately 90º of flexion.

Preferred leg was established by kicking a ball three times; the leg used for kicking was considered the preferred leg (100% of children kick the ball with the same time in every trial).

Protocol SLL (key points)

1. **Warm-up**: standardized oriented warm-up.
   - Hip, knee and ankle joints mobility.
   - High amplitude knee flexion-extension x 5.
   - Skipping x 10”.
   - Countermovement jump x 5.
2. **Practice SLL**: 3 trials per height and leg (around 18 trials).
3. **Hands location**: elbow flexed with arms located at the hip under the superior iliac crest.
4. **Leg of landing**: as natural as possible with the preferred leg.
5. **Free leg**: 90º knee flexion.
6. **Type of leg**: preferred vs non-preferred.
   - It requires kicking a ball (the kick leg is considered the preferred kick).
7. **Shoes**: barefoot.
8. **Rest time between trials**: the time needed for the next trial (around 1-2 min).
9. **Take off platform location**: 5cm from the rear side of the force platform.
10. **Onset movement**: A step with the land leg (preferred).

**Onset condition**: On the take of platform, stand with hands at the hip under the superior iliac crest and both legs separated at shoulder width.

The **number of trials** (accepted and nor accepted) was counted in order to get indirect observed performance estimation.

**Accepted trial was considered with the existence of some criteria:**

1. Flight phase exists (no contact with the height and force platform exists simultaneously).
2. No jump exists (no ascendant vertical movement is seen in markers during drop).
3. After landing (next 10s), no steps exists (GRF in z axes appears every time when markers are revised).
4. During 10s balance, no contact with the height platform exists (foot of the free leg does not touch the height platform).
5. During 10s balance, monopodal balance exists (no double contact exists, only a foot is in contact with the ground).
